# European practice on neurovascular late effects after pediatric radiotherapy: considerations on dose planning and follow-up – a HARMONIC/SIOPE ROWG survey

**DOI:** 10.2340/1651-226X.2025.43028

**Published:** 2025-05-22

**Authors:** Laura Toussaint, Louise Tram Henriksen, Karen Van Beek, Stéphanie Bolle, Charlotte Demoor-Goldschmidt, Jenny Gains, Morten Høyer, Geert O. Janssens, Rolf-Dieter Kortmann, Catia Martins Pedro, Beate Timmermann, Katrin Scheinemann, Yasmin Lassen-Ramshad

**Affiliations:** a Danish Centre for Particle Therapy, Aarhus University Hospital, Aarhus, Denmark; b Centre for Paediatric and Adolescent Cancer, Department of Paediatric and Adolescent Medicine, Aarhus University Hospital, Aarhus, Denmark; c Department of Radiation Oncology, University Hospital Leuven, Leuven, Belgium; d Institut Gustave Roussy, Department of Radiation Oncology, Villejuif, France; e Cancer and Radiation Team, INSERM UMRS 1018, Villejuif, France; f Paediatric oncology and hematology, University Hospital of Caen, Caen, France; g Paediatric oncology and hematology, University Hospital of Angers, Angers, France; h Department of Oncology, University College London Hospitals NHS Foundation Trust, London, UK; i Department of Radiation Oncology, University Medical Center Utrecht, Utrecht, The Netherlands; j Princess Maxima Center for Pediatric Oncology, Utrecht, The Netherlands; k Department of Radiation Oncology, University of Leipzig, Leipzig, Germany; l Radiotherapy Department, Instituto Português de Oncologia Francisco Gentil, Centro de Lisboa, Lisbon, Portugal; m West German Proton Therapy Centre (WPE), Essen University Hospital, Essen, Germany; n West German Cancer Centre (WTZ), German Cancer Consortium (DKTK), Essen, Germany; o Division of Oncology/ Hematology, Children’s Hospital of Eastern Switzerland, St. Gallen, Switzerland; p Faculty of Health Sciences and Medicine, University of Lucerne, Lucerne, Switzerland

**Keywords:** Pediatrics, CNS tumors, neurovascular, late effects, radiotherapy, survey, European practice

## Abstract

**Background and purpose:**

The risk of developing neurovascular late effects after radiotherapy is an area of concern when treating pediatric brain tumor patients. However, knowledge is sparse regarding best practice for clinical management during the radiotherapy (RT) planning process and follow-up examinations. This study therefore aimed at mapping how the risk of neurovascular late effects is considered for pediatric brain or skull base tumor patients treated with radiotherapy in Europe.

**Materials and methods:**

Two web-based surveys ‑ a RT and a pediatric oncology (PO) survey - were distributed to members of the SIOPE radiotherapy working group or PANCARE and SIOPE brain tumor group, respectively.

**Results:**

The RT survey was completed by 47 participants from 18 different European countries and the PO survey by 33 participants (mostly pediatric (neuro)oncologists) from 15 countries. Overall, the answers highlighted that neurovascular late effects are currently not well included in European clinical practice, neither at the time of radiotherapy nor in the follow-up process.

**Interpretation:**

There is a need for raising general awareness about the topic, as well as for potential risk-stratified prevention measures and follow-up guidelines.

## Introduction

Radiation-induced neurovascular disease, presenting as either large- or small-vessel disease, is a well-documented late effect in pediatric brain tumor survivors [1]. The Pediatric Normal Tissue Effects in the Clinic (PENTEC) collaboration recently performed a meta-analysis on the risk of neurovascular toxicity after cranial irradiation of pediatric patients [2]. This work highlighted that long-term survivors of pediatric brain tumors are at an increased risk of developing neurovascular disease compared to baseline risk, with attained age and prescribed radiotherapy (RT) dose playing a major role.

While this analysis was mostly based on single-center data with relatively small cohorts of patients treated with older RT techniques, the HARMONIC protocol is prospectively studying the incidence of neurovascular events following modern RT delivery modalities [3]. In this European multicenter registry, participating institutions are aligning their follow-up magnetic resonance imaging (MRI) protocols (sequences and timing) to investigate the association between early vascular changes seen on MRI, radiation dose to neurovascular organs at risk (OAR), and the development of neurovascular late effects.

However, outside of such studies where practices are controlled and aligned, it remains unclear what the current clinical practice is across European centers in terms of screening, risk reduction, but also recommendations and patients’ information about neurovascular late effects.

The aim of this project was therefore to map how the risk of neurovascular late effects is considered when treating pediatric patients with a brain or skull base tumor with RT, both in clinical RT routine and in follow-up examinations.

## Materials and methods

Two web-based surveys were prepared in the frame of the HARMONIC project (harmonicproject.eu), together with the SIOPE Radiation Oncology Working Group (ROWG). Access links to the surveys were subsequently distributed via the mailing list of the ROWG (radiotherapy survey, RT) or the PANCARE and SIOPE brain tumor working group (pediatric oncology survey, PO). Data collection ran from December 2023 to February 2024. Only one respondent per institution was needed as we were interested in center-wise experience.

The RT survey included 33 questions and the PO survey 38 questions, both for a completion time of around 15 min. Both surveys investigated screening and information of the patients, recommendations, and follow-up programs. The RT survey included additional questions on neurovascular OARs and associated dose constraints. Proposed large vessel disease OARs included the Circle of Willis, its individual arteries, the middle cerebral artery, and surrogate structures (i.e. optic chiasm, prepontine cistern, or suprasellar cistern) [2, 4]. For small vessel disease, the whole brain was proposed [5]. For both categories, a free text option was also available for respondents to report on other potential OARs. For the PO survey, a case study was proposed with four scenarios: how would the respondent manage a symptomatic vs. asymptomatic patient presenting with large vs. small vessel disease newly observed on MRI? For those cases, the explored categories included referral, medical treatment, lifestyle recommendations, screening of neurocognitive function, and future follow-up.

Practical details about the survey design, focusing on items from the CHERRIES guidelines for reporting on web-based surveys [6], are reported in the Supplementary Material. Due to a rather low number of respondents, the results are presented as descriptive; therefore, no statistical correction was applied to adjust for the potentially nonrepresentative sample, and no attempt to calculate a participation or completion rate was done.

## Results

### RT survey

Forty-seven participants from 18 European countries completed the RT survey (Supplementary Figure 1), with five institutions reporting having a research focus on neurovascular late effects. Of all respondents, 66% (31/47) had more than 10 years of experience in treating pediatric patients with brain or skull base tumors with RT. The available treatment modalities are reported in Supplementary Figure 2.

Overall, 89% (42/47) of the respondents never delineated specific OARs for small vessel disease, and 83% (39/47) never delineated specific OARs for large vessel disease. The main reasons were that those OARs are not traditionally integrated into clinical protocols (28/39) and have no dose constraints (31/39). Further information about neurovascular OARs and dose constraints is detailed in Supplementary Figure 3 and Supplementary Figure 4, respectively.

**Figure 1:**
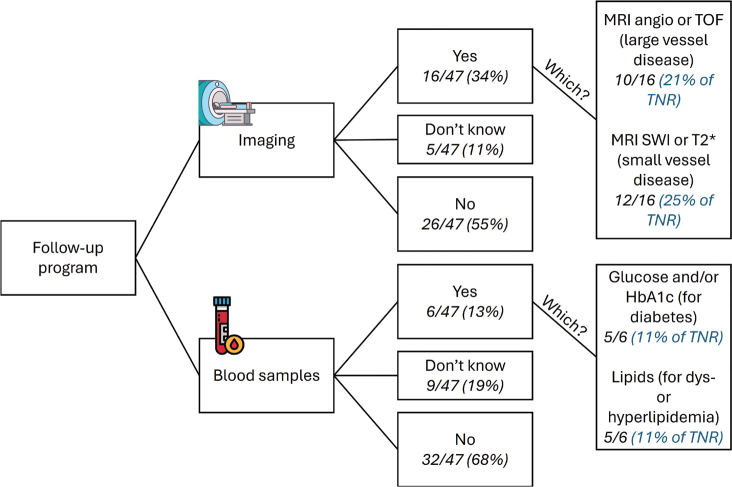
Answers on the existence of a follow-up program (being institutional, national, or together with the pediatric oncology/neurology department) for neurovascular late effects after radiotherapy (RT) from the RT survey. Angio: angiography; TOF: time of flight; SWI: susceptibility weighted imaging; HbA1c: glycated hemoglobin; TNR: total number of responders.

Practices for screening, patient information, and recommendations are summarized in Table 1. Briefly, of the 12/47 (26%) respondents performing some screening of neurovascular disease before RT, 10/12 asked about family history of neurovascular disease, 8/12 performed MRI with neurovascular sequences, and 9/12 assessed comorbidities such as hypertension, diabetes, or hyperlipidemia. Of 21/47 (45%) respondents giving specific recommendations to their patients, 18 had advice entailing lifestyle (i.e. no smoking, healthy diet, and regular physical exercise). Recommendations given to the treating pediatric oncologists were about including neurovascular MRI sequences (e.g. angiography, T2*) at follow-up, with timing varying from yearly to every five years (13/21). Most respondents did not have a specific follow-up program (being institutional or national) for neither imaging (55%) nor blood sampling (68%), as detailed in Figure 1.

**Table 1:** Summary of the proportion of respondents that screened, informed, and made recommendations regarding the risk of neurovascular late effects to the patients and their parents, for both surveys

Clinical procedure	Group considered	RT survey (before RT)	PO survey (in follow-up)
**Screening** for neurovascular disease (both large and small) *in the PO survey, screening is reported separately for MRI and blood sampling.	No patients	35/47 74%	18/33 55%
	All patients	3/47 6%	*MRI* 12/33 40% *Blood Sampling* 10/33 30%
	Some patients	NF1 6/47 13% Suprasellar RT 4/47 8%	*MRI* Symptom suspicion 4/33 12% *Blood Sampling* CP 2/33 6%
**Informing** of the risk of ***large vessel disease***	No patients	7/47 15%	0/33 0%
	All patients	22/47 47%	22/33 67%
	Some patients	CP 12/47 25%	NF1 9/33 27% CP 8/33 24%
**Informing** of the risk of ***small vessel disease***	No patients	12/47 27%	2/33 6%
	All patients	24/47 56%	25/33 81%
	Some patients	MB 7/47 16%	NF1 5/33 15% RO recommendation 5/33 15%
**Recommendations** if the patient is thought to be at risk of neurovascular disease	No recommendations	26/47 55%	*Large vessel disease* 20/33 61% *Small vessel disease* 22/33 67%
	Most common recommendations	Healthy diet, no smoking, regular physical activity	

CP: craniopharyngioma, MB: medulloblastoma, NF1: neurofibromatosis type I, RT: radiotherapy, PO: pediatric oncologist, RO: radiation oncologist. Note that in some cases (especially when answering ‘for some patients’), respondents could select several options. Therefore, the sum of percentages presented in the table does not necessarily correspond to the total number of respondents.

### PO survey

Thirty-three respondents from 15 different European countries participated in the PO survey (Supplementary Figure 1), with only three institutions having a specific research focus on neurovascular late effects. Most of the respondents were pediatric oncologists (15/33, 45%) or pediatric neuro-oncologists (12/33, 36%). Of all respondents, 73% (24/33) had more than 10 years of experience in working with pediatric patients having received RT for a brain or skull base tumor.

**Figure 2:**
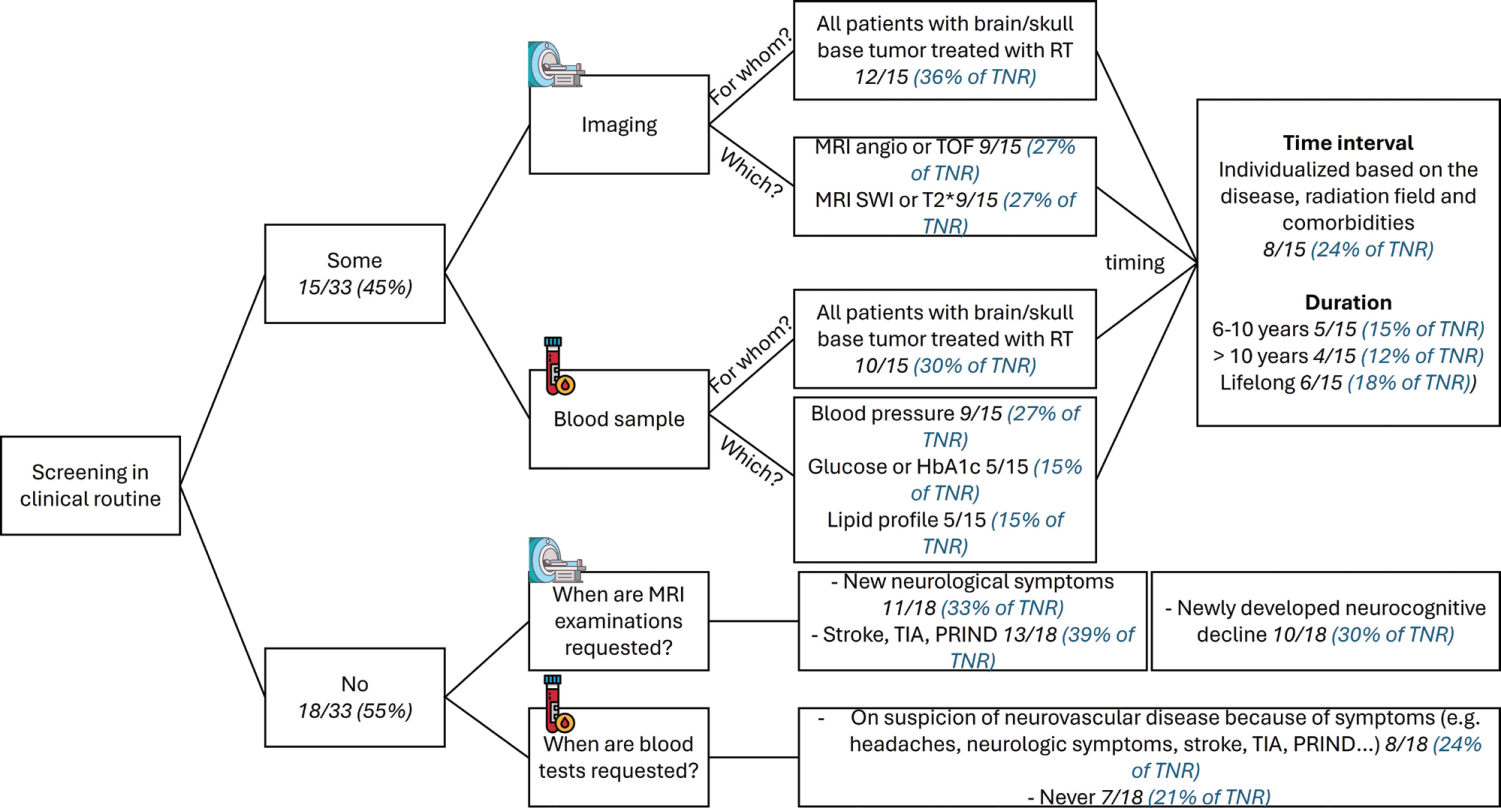
Current routine practices for screening (imaging and blood sampling) of neurovascular late effects after RT from the PO survey. angio: angiography; TOF: time of flight; SWI: susceptibility weighted imaging; HbA1c: glycated hemoglobin; TIA: transient ischemic attack; PRIND: prolonged reversible ischemic neurologic deficit; TNR: total number of responders.

Practices for screening, patient information, and recommendations are summarized in Table 1 and Figure 2. From all respondents, 67% (22/33) informed all patients of the risk of developing large vessel disease and 81% (25/33) of the risk of small vessel disease. In terms of recommendations, similar advice as in the RT survey was given for reducing the risk of large or small vessel disease, mostly focusing on lifestyle with for example healthy diet, no smoking, regular physical activity, and symptom alertness. Overall, 73% of the respondents (24/33) did not have guidelines (being national or institutional) for follow-up regarding neurovascular large or small vessel disease after brain/skull base RT in childhood.

Results from the case studies are reported in Supplementary Table 1. In the four cases, the same lifestyle recommendations would be given and would again entail a healthy diet, no smoking, and regular physical activity, especially for patients at risk for large vessel disease (62% in asymptomatic cases and 66% in symptomatic cases). For the two cases of symptomatic patients, when medical treatment was advised by the PO (i.e. in 39 and 28% of the responses for large and small vessel disease symptoms, respectively), the treatment would be based on recommendations from the neurologist. However, in most cases, starting medical treatment was highly patient specific, as indicated by the most common answer being ‘maybe’.

## Discussion

The outcome of these survey demonstrated that the risk of neurovascular late effects is currently not well included in clinical practice of European pediatric RT centers. Similarly, neurovascular screening after RT for pediatric brain tumors was performed in less than 50% of the responding institutions with only a few institutions having specific guidelines.

In the RT survey, 83% of the respondents never delineated neurovascular OARs for large-vessel disease risk. While the lack of dose constraints was the main cited reason, 46% of the respondents reported a lack of consensus on OARs and delineation atlas. To overcome those challenges, a list of relevant OARs for the risk of large-vessel disease has been proposed in the frame of the HARMONIC project, together with a delineation atlas [4]. In this atlas, the suprasellar cistern was also proposed as a surrogate for the dose to the Circle of Willis. The PENTEC report further recommended that delineation of surrogate structures (i.e. optic chiasm or suprasellar cistern) be performed ‘at a minimum’ in clinical routine [2].

The main argument in favor of using a surrogate structure was that the delineation process would not be too time consuming (with time also cited as a barrier to neurovascular OAR delineation in our survey), thereby ensuring compatibility with clinical practice. However, the validation of the dose to the suprasellar cistern as a surrogate for dose to the Circle of Willis was only done for proton therapy dose plans [4]. The applicability to photon therapy plans should therefore also be investigated. Indeed, having the same OAR for dose surrogate, independent of treatment modality, would help ensure broader acceptance by the RT community. Furthermore, the structure has not yet been validated in a clinical context, and studies of clinical outcomes in this patient’s population will therefore be required.

In their recommendations for the management of childhood cancer survivors at risk of stroke after cranial irradiation, Keney et al. reported full consensus on the need for MRI surveillance in patients with a history of small or large vessel changes on prior imaging, even if asymptomatic [7]. However, no consensus was reached for asymptomatic patients without prior history. Some of the arguments against systematic MRI screening were the risk of imaging findings with unclear clinical significance, with the associated anxiety to the patient, as well as the cost of such a follow-up program. In the present survey, only 36% of respondents to the PO-survey performed MRI surveillance for all brain tumor patients treated with RT to screen for neurovascular late effects, potentially highlighting similar considerations.

By developing and implementing common follow-up guidelines, data on risk factors of neurovascular late effects after treatment of a pediatric brain tumor will be generated, allowing for the definition of risk groups. Besides increasing awareness on these potential late effects, clinical studies with collaborations across centers are required to advance knowledge about neurovascular late effects.

Some limitations must be recognized: while the analysis was based on the CHERRIES guidelines for reporting of internet surveys [6], the development of the questions was not. In addition, such web-based surveys can be subject to considerable bias resulting from the nonrepresentative nature of the responders and the self-selection aspect relating to them, where responders to this survey are more likely to be interested in neurovascular late effects. Therefore, the collected answers could be biased.

However, in our study, even if our responders would be a group particularly aware of the risk of neurovascular late effects after RT, it did not translate into their clinical practice. Indeed, most responders did not have specific practice for neurovascular late effects. Our results would therefore likely apply to the European community in a larger sense: neurovascular late effects are very rarely included in clinical practice of both radiation oncology and PO across European centers.

Both surveys reflected the view of a relatively small sample compared to the total number of members on the email lists used for distribution. This remark was especially true for the PO survey. However, as described above, it is unlikely that the nonresponders would be the ones with, for example specific guidelines or risk-management strategies for neurovascular late effects. In addition, opinions from various countries were collected, reflecting different practices. Again, our results could most likely be descriptive of the European community at large.

Overall, consideration of neurovascular late effects is currently not well included in European pediatric clinical practice. As suggested in the PENTEC report [2], there is need for more data to assist clinical practitioners in terms of screening, informing, recommendations, and follow-up guidelines for pediatric brain tumor patients. A collaborative effort towards gathering further evidence would improve understanding of risk factors of neurovascular late effects and allow for the development of risk-stratified follow-up guidelines for improved survivorship care in this patient’s population.

## Supplementary Material



## Data Availability

This study was based on the results from two surveys. Data supporting the findings of this study are available from the corresponding author upon reasonable request.
