# Supplementary material for: European practice on neurovascular late effects after pediatric radiotherapy: considerations on dose planning and follow-up – a HARMONIC/SIOPE ROWG survey
Source: Acta Oncol. 2025 May 22;64:43028. doi: 10.2340/1651-226X.2025.43028 (PMC12128628; doi:10.2340/1651-226X.2025.43028)
Supplement: Supplementary file 1 [file AO-64-43028-s1.pdf]

Supplementary material has been published as submitted. It has not been copyedited, or typeset by Acta Oncologica

Supplementary Material

Survey Design:

The surveys were developed by three members of the investigator's team after a review of the literature on neurovascular late effects after RT in children. The surveys were then discussed, reviewed, edited and pre-tested by the other members of the team, ensuring completeness of the questions and functionality of the online platform. Data was collected through the online platform SurveyXact, offering tools for automatic capturing of the answers and password-protection of the results. Participation in the surveys was voluntary and could be anonymous, with only the institution name being mandatory. The questions' order was not randomized, and adaptive questioning was used when follow-up questions were required based on previous answers. Respondents could review their answers by using the back button, however it was not possible to modify them after submission. Completeness check was done automatically by the platform, highlighting missing answers when mandatory items were not completed and preventing the respondents from moving to the next questions. All items, when applicable, had an alternative 'other' option with associated text field. Only complete questionnaires were included in the final analysis, and it was manually ensured that the same individual did not submit multiple questionnaires. Due to a rather low number of respondents, the results are presented as descriptive; therefore, no statistical correction was applied to adjust for the potentially non-representative sample, and no attempt to calculate a participation or completion rate was done.

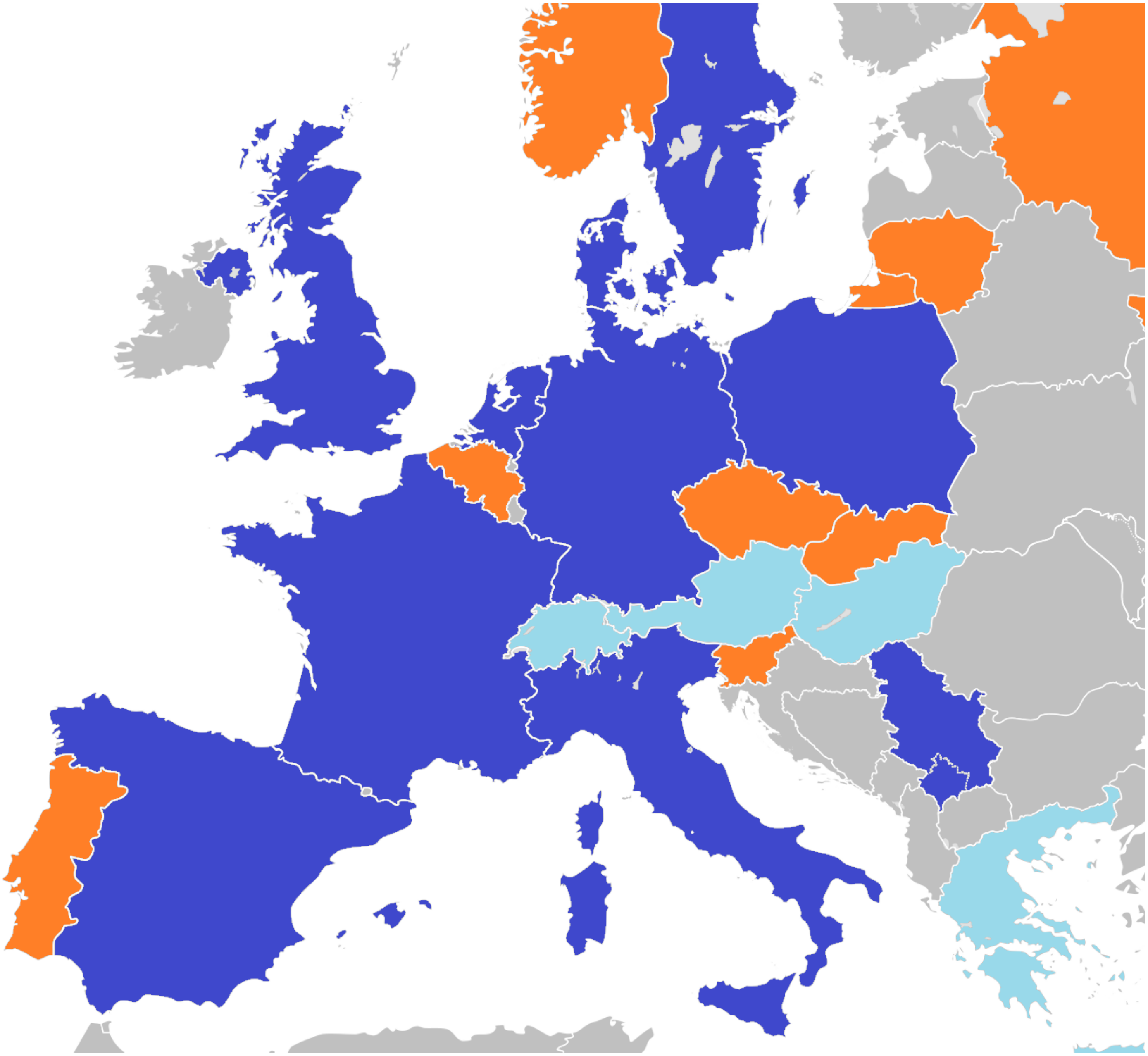

Supplementary Figure 1: Map of European countries represented in the received answers. The orange countries responded to the RT survey only, the light blue countries to the PO survey only, and the dark blue countries to both surveys.

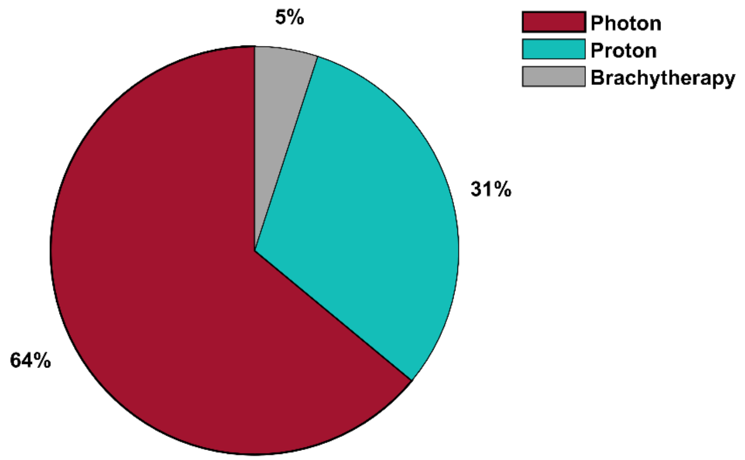

Supplementary Figure 2: Pie chart of the treatment modalities available to the respondents of the RT-survey.

Figures

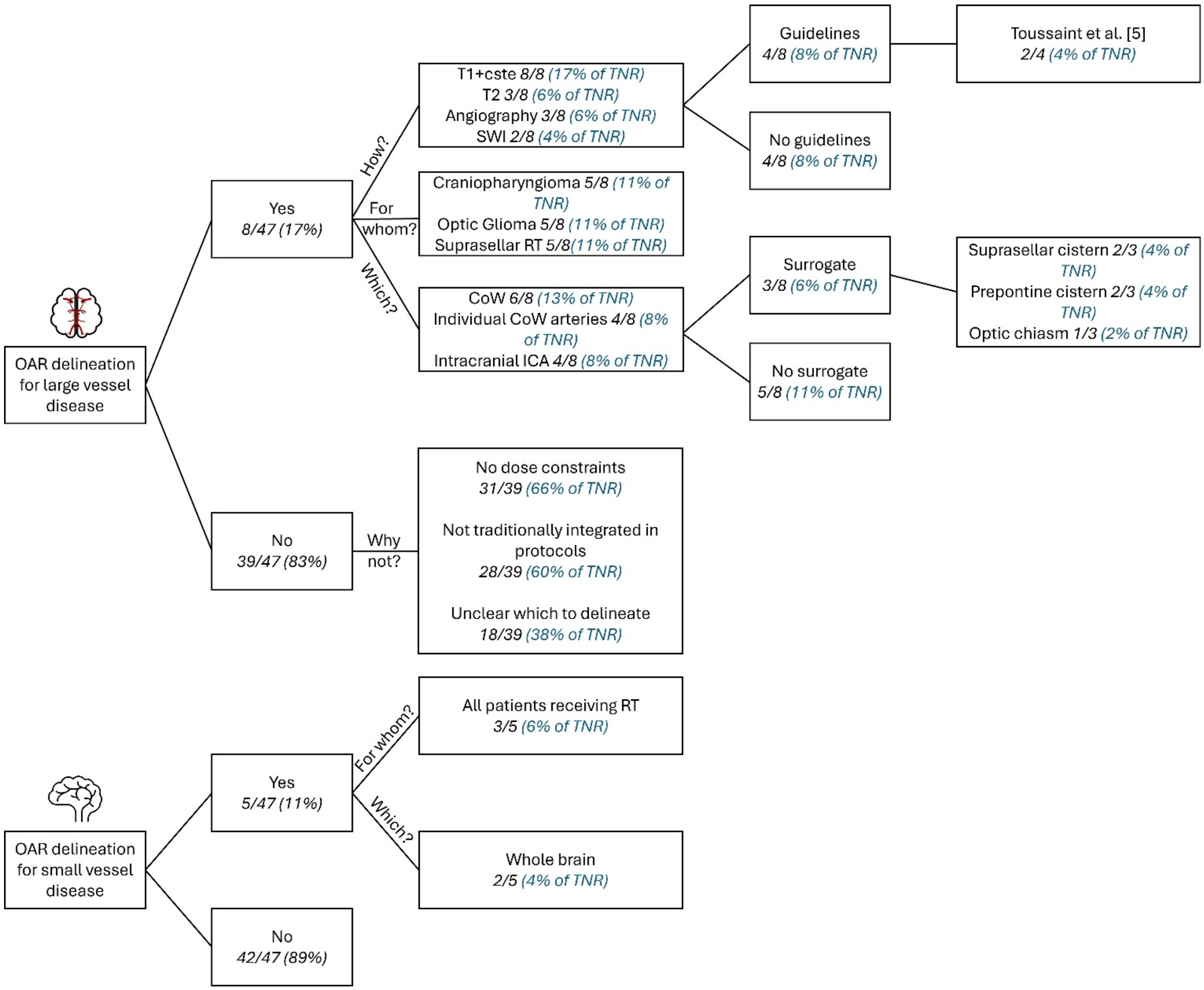

Supplementary Figure 3: Answers on the delineation of organs at risk for neurovascular large and small vessel disease from the RT-survey. Abbreviations: OAR organs at risk, CoW Circle of Willis, CSTE contrast, SWI susceptibility weighted imaging, RT radiotherapy, ICA internal carotid artery, TNR: total number of responders.

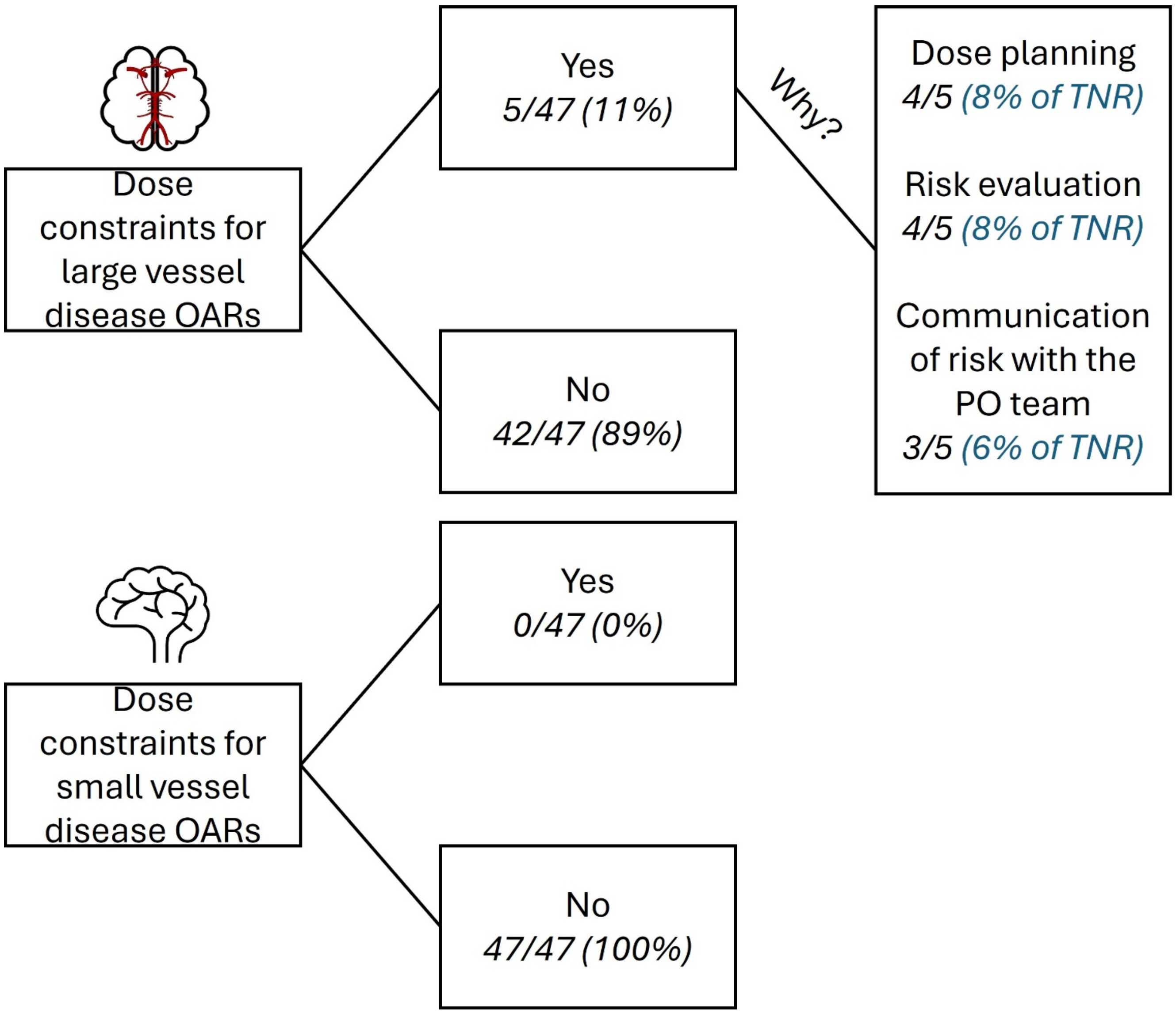

Supplementary Figure 4: Answers on the dose constraints items for neurovascular organs at risk from the RT-survey.

Abbreviations: OAR organs at risk, PO pediatric oncologist, TNR: total number of responders.

Supplementary Table 1: Results of the four cases submitted in the PO survey. NB: not all 33 participants answered those questions. LVD: large vessel disease; SVD: small vessel disease, MRI magnetic resonance imaging.

The patient will:	Be referred to a neurologist or neurosurgeon	Start some form of medical treatment	Receive specific lifestyle recommendations	Be screened for neurocognitive dysfunction	Be followed in the future for neurovascular disease
**Case 1**<br>Large Vessel Disease on MRI, asymptomatic	Yes<br>***32/33 (97%)***	Yes<br>***3/33 (9%)***<br><br>Maybe<br>***21/33 (64%)***	Yes<br>***18/29 (62%)***	Yes<br>***22/31 (71%)***	- As recommended by the neurologist or neurosurgeon<br>***26/32 (81%)***<br>- Individual follow-up<br>***14/32 (44%)***<br>- Regular screening for hypertension, obesity, diabetes, dys- or hyperlipidemia<br>***12/32 (38%)***
**Case 2**<br>Small Vessel Disease on MRI, asymptomatic	Yes<br>***23/33 (70%)***	Yes<br>***4/32 (13%)***<br><br>Maybe<br>***15/32 (47%)***	Yes<br>***14/30 (47%)***	Yes<br>***20/31 (65%)***	- As recommended by the neurologist or neurosurgeon<br>***16/31 (52%)***<br>- Individual follow-up<br>***14/31 (45%)***<br>- Regular screening for hypertension, obesity, diabetes, dys- or hyperlipidemia<br>***8/31 (26%)***
**Case 3**<br>Large Vessel Disease on MRI, symptomatic	Yes<br>***32/33 (97%)***	Yes<br>***12/31 (39%)***<br><br>Maybe<br>***15/31 (48%)***	Yes<br>***19/29 (66%)***	Yes<br>***22/30 (73%)***	- As recommended by the neurologist or neurosurgeon<br>***23/32 (72%)***<br>- Individual follow-up<br>***16/32 (50%)***<br>- Regular screening for hypertension, obesity, diabetes, dys- or hyperlipidemia<br>***11/32 (34%)***
**Case 4**<br>Small Vessel Disease on MRI, symptomatic	Yes<br>***30/33 (91%)***	Yes<br>***9/32 (28%)***<br><br>Maybe<br>***20/32 (63%)***	Yes<br>***16/30 (53%)***	Yes<br>***25/31 (81%)***	- As recommended by the neurologist or neurosurgeon<br>***23/32 (72%)***<br>- Individual follow-up<br>***15/32 (47%)***

					- Regular screening for hypertension, obesity, diabetes, dys- or hyperlipidemia<br>***11/32 (34%)***
